# Assessing the DNA Damaging Effectiveness of Ionizing Radiation Using Plasmid DNA

**DOI:** 10.3390/ijms232012459

**Published:** 2022-10-18

**Authors:** Yara Maayah, Humza Nusrat, Geordi Pang, Mauro Tambasco

**Affiliations:** 1 Department of Physics, San Diego State University, San Diego, CA 92182, USA; 2 Odette Cancer Centre, Department of Radiation Oncology, University of Toronto, Toronto, ON M4N 3M5, Canada

**Keywords:** DNA-based detector, X-ray ionizing radiation, DNA DSB damage, oxidized DNA base damage, linear energy transfer, relative biological effectiveness

## Abstract

Plasmid DNA is useful for investigating the DNA damaging effects of ionizing radiation. In this study, we have explored the feasibility of plasmid DNA-based detectors to assess the DNA damaging effectiveness of two radiotherapy X-ray beam qualities after undergoing return shipment of ~8000 km between two institutions. The detectors consisted of 18 μL of pBR322 DNA enclosed with an aluminum seal in nine cylindrical cavities drilled into polycarbonate blocks. We shipped them to Toronto, Canada for irradiation with either 100 kVp or 6 MV  X-ray beams to doses of 10, 20, and 30 Gy in triplicate before being shipped back to San Diego, USA. The Toronto return shipment also included non-irradiated controls and we kept a separate set of controls in San Diego. In San Diego, we quantified DNA single strand breaks (SSBs), double strand breaks (DSBs), and applied Nth and Fpg enzymes to quantify oxidized base damage. The rate of DSBs/Gy/plasmid was 2.8±0.7 greater for the 100 kVp than the 6 MV irradiation. The 100 kVp irradiation also resulted in 5±2 times more DSBs/SSB than the 6 MV beam, demonstrating that the detector is sensitive enough to quantify relative DNA damage effectiveness, even after shipment over thousands of kilometers.

## 1. Introduction

Approximately 50% of all cancer cases in the U.S. are treated with radiation therapy [1]. The guiding principle of radiation therapy is to maximize tumor cell death while sparing healthy tissues to decrease mortality and morbidity [2]. In terms of external beam photon radiotherapy, the bulk of sparsely ionizing X-ray energy is expended along the tracks of the ionized electrons [3]. These electron tracks can vary in linear energy transfer (LET), the amount of energy deposited per unit track length traveled by charged particles [3]. The distribution of electron tracks within cells can cause direct damage to cellular components, as well as indirect damage through the production of free radicals, predominantly hydroxyl radicals. Of the cellular components damaged, DNA has been shown to be the principal cellular target of radiotherapy, leading to reproductive cell death [4,5]. The types of damage induced to cellular DNA investigated in this study include single strand breaks (SSBs), double strand breaks (DSBs), isolated and clustered heat labile sites (IHLS and CHLS, respectively) and isolated and clustered oxidized base damage (IOBD and COBD, respectively) [6]. As the amount and complexity of DNA damage increases, cells have increasing difficulty repairing the DNA, leading to cell death via apoptosis, mitotic catastrophe, autophagy, or necrosis [6,7].

Absorbed radiation dose, the amount of energy deposited per unit mass, is the quantity used to describe the amount of ionizing radiation needed to treat a tumor target volume [8]. However, absorbed dose alone is not an adequate index of the DNA damaging capacity and the resulting biological effect of ionizing radiation. That is, for the same absorbed dose, the DNA damage and biological outcome of ionizing radiations with different LET will vary. Furthermore, current dosimeters used to measure absorbed dose in radiation therapy applications cannot account for LET effects on DNA damage and eventual biological outcome. Although phenomenological models that relate the dose-averaged linear energy transfer (LET_D_) can be used as a representative quantity for the cumulative biological effectiveness of radiation delivery of high LET beams [9,10,11,12], they have limitations [13] and the implementation of such computational models would benefit from quality control measurements. Hence, a sufficiently sensitive DNA-based dosimeter is needed to assess the DNA damaging effects of spatially varying LET_D_.

Recent studies have investigated the use of DNA-based dosimeters to measure the DNA damaging effectiveness of ionizing radiation. Some of these studies investigated the use of linear DNA, having one biotinylated end attached to magnetic streptavidin beads and the other attached to a fluorescent dye to measure DNA DSB damage induced by ionizing radiation [14,15,16,17]. One of these studies compared the DNA DSB damage of a 160 kVp X-ray beam versus a 6 MV X-ray beam [14], and another compared DSB damage of a 160 MeV proton beam to a 6 MV photon beam [15]. These studies were both able to detect differences in DNA DSB damage due to LET differences in the beams. However, the limitations of the linear DNA-based dosimeter approach used in these studies is that it can only measure DSB DNA damage and not SSB or oxidized base damage that contribute to complex clustered DNA damage. Moreover, this approach currently lacks precision, and it does not quantify instances in which more than one DSB has occurred.

An alternative, older approach to investigate DNA damage quantifies the conformational changes of supercoiled DNA when it is exposed to ionizing radiation [18,19]. That is, the supercoiled DNA conformation relaxes to an open circular or linear conformation following a SSB or DSB, respectively. Plasmid DNA is advantageous in studying ionizing radiation induced DNA damage as it possesses three conformations: supercoiled, open circular, and linear corresponding to no damage, SSB, and DSB damage, respectively. These SSB and DSB conformations can be quantified by separating them into distinct band intensities using gel electrophoresis. Furthermore, IOBD and COBD can also be quantified by applying enzymatic treatments to the DNA, which effectively converts IOBD and COBD to SSB and DSB damage, respectively [20,21]. Previous studies using this approach successfully quantified DNA damage as a function of LET differences [22,23,24].

In this study, we designed and constructed a transportable DNA detector based on the above DNA conformational modifying assay to attain a more comprehensive assessment of DNA damage by quantifying SSBs, DSBs, IHLS, CHLS, IOBD, and COBD caused by 100 kVp and 6 MV X-ray beams. We used our plasmid DNA-based detector to test the feasibility of return shipment across the U.S./Canadian border and transportation over ~8000 km. We also demonstrated the ability of the plasmid DNA-based detector to quantify the DNA damaging effectiveness of ionizing radiations possessing small LET differences arising from the same type of ionizing radiation (photons) of different energies (100 kVp and 6 MV). This quantification was carried out in terms of the number of DSBs/Gy/plasmid and the proportion of DSBs per SSB quantified for the several types of DNA damage, which we have defined as the DNA damaging effectiveness (DDE).

## 2. Results

### 2.1. Effects of Shipping on the Plasmid DNA Conformations

We measured and compared all plasmid DNA conformational changes in the control sent to Toronto with the control kept in San Diego (Table 1). To account for all shipping effects on the DNA, we measured these conformational changes for non-incubated and non-enzymatically treated DNA as well as DNA that was incubated for one hour, and DNA that was incubated while being treated with Fpg and Nth enzymes for one hour. Table 1 shows that the DNA conformational changes due to shipping were almost exclusively to the open circular conformation.

Table 2 summarizes all the types of plasmid DNA damage that could be deduced from the conformational changes shown in Table 1. From Table 2, we see that the DNA shipped to Toronto and back to San Diego incurred more SSBs, IHLS, and IOBD than the control DNA kept in San Diego. However, it is important to note that the more severe type of damage (i.e., DSB, CHLS, and COBD) was either zero or negligible compared to the measurement uncertainty. It is also worth noting that although we measured the plasmid DNA that remained in San Diego to be within the manufacturer’s specification of ~95% supercoiled (Table 1), we did find this DNA to have significant IOBD (Table 2).

**Figure 1:**
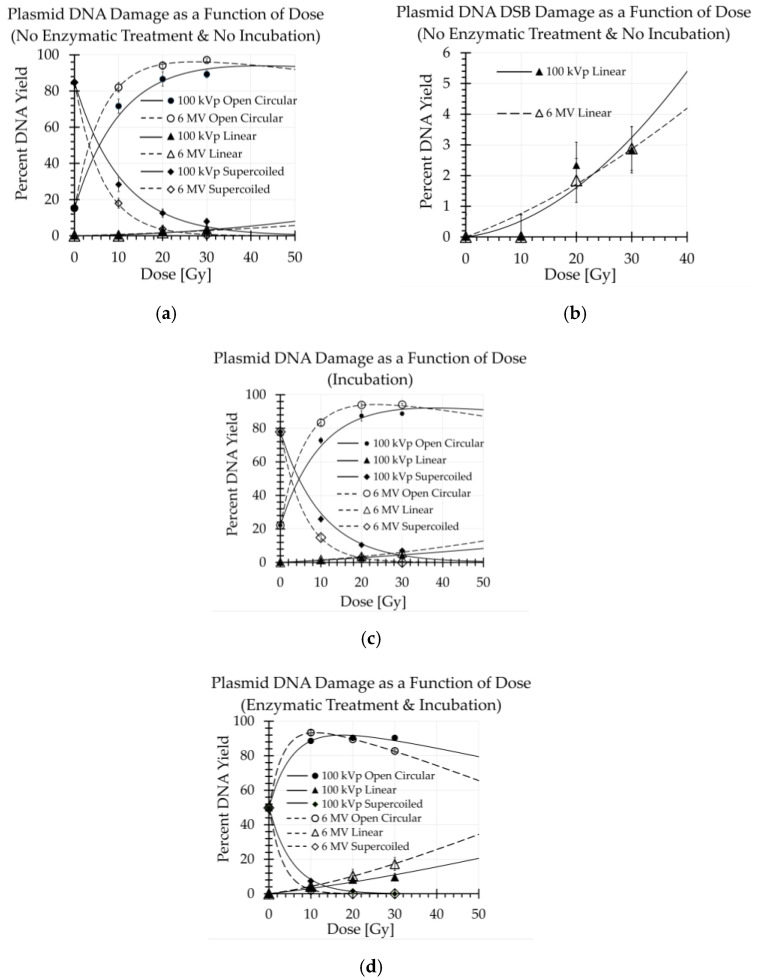
The measured yields at 0, 10, 20, and 30 Gy are plotted and the McMahon and Currell robust plasmid DNA curve fits of the data are displayed for (**a**) control and irradiated samples, (**b**) a magnified view of the linear DNA conformation as a function of dose shown in (**a**), (**c**) samples incubated post irradiation, and (**d**) samples incubated with enzymatic (Nth and Fpg) treatment post irradiation [25].

The damage due to shipping was mostly likely due to the temperature changes (going between −20 °C in freezer storage and ranging from 0 to 8 °C during the two-day FedEx transit time that the Styrofoam shipping container with the DNA-based detectors on ice packs travelled each way). Despite this damage, it should be noted that the amount of degradation of the supercoiled DNA due to shipping was acceptable, as there was still an observable supercoiled DNA signal up to the maximum absorbed radiation dose of 30 Gy used in this study (Figure 1a).

### 2.2. Yields of SSBs and DSBs/Gy/Plasmid of Irradiation

We measured the amount of open circular conformation induced by the 6 MV beam to be 1.18, 1.10, and 1.11 times greater than the 100 kVp beam at doses of 10, 20, and 30 Gy, respectively (Figure 1a). Hence, there was a 10% to 18% greater induction of SSBs in the 6 MV versus the 100 kVp beam. After incubation alone, the measured percent yields of DSBs from 10 to 30 Gy, representing clustered heat labile sites (CHLS), were 20% to 30% greater for the 6 MV than the 100 kVp beam (Figure 1c). After enzymatic treatment, the measured percent yields of DSBs, representing clustered oxidized base damage (COBD), were 8% to 80% greater for the 6 MV than the 100 kVp beam (Figure 1d). Hence, these results show that significantly more CHLS and COBD damage occurred in the lower LET 6 MV beam than the higher LET 100 kVp beam. Furthermore, the differences in clustered base damage between these two beams increased with dose.

**Figure 2:**
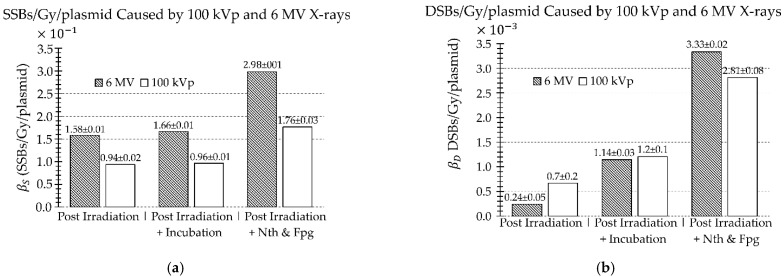
Comparison of the rates of (**a**) SSBs/Gy/plasmid and (**b**) DSBs/Gy/plasmid, caused by the 100 kVp versus 6 MV beam as measured post irradiation, post irradiation and incubation, and post irradiation and enzymatic treatment. Uncertainties represent 68% confidence intervals.

Figure 2 shows the probability parameters $\beta_{S}$ and $\beta_{D}$, representing the number of SSBs and DSBs per Gy per plasmid, respectively, that we computed from fits of the measured data. There was approximately a 1.7-, 1.8-, and 2.1-times greater number of SSBs/Gy/plasmid caused by the 6 MV than the 100 kVp beam post irradiation, post irradiation incubation with no enzymatic treatment, and post irradiation incubation and enzymatic treatment, respectively (Figure 2a). For DSBs, there was 2.8 times greater number of DSBs/Gy/plasmid in the 100 kVp than the 6 MV beam after irradiation (Figure 2b). Finally, there was 1.2 times greater number of DSBs/Gy/plasmid occurred in the 6 MV beam than the 100 kVp beam after enzymatic treatment with incubation was applied and there was negligible difference between the number of DSBs/Gy/plasmid when incubation with no enzymatic treatment was applied (Figure 2b). The difference in $\beta_{S}$ and $\beta_{D}$ parameters between post irradiation with incubation alone versus incubation with enzymatic treatment is indicative of IOBD and COBD/Gy/plasmid, respectively, as quantified in Table 3.

### 2.3. Heat Labile Sites and Isolated and Clustered Oxidized Base Damage

The 6 MV beam irradiation resulted in greater amounts of SSBs, IHLS, CHLS, IOBD, and COBD per Gy/plasmid than the 100 kVp beam (Figure 2 and Table 3). However, as noted above, the 100 kVp beam irradiation produced a greater number of DSBs/Gy/plasmid than the 6 MV beam. The number of IHLS was quite small, contributing only a factor of 1.03±0.02 and 1.05±0.01 more SSBs/Gy/plasmid in the 100 kVp beam and 6 MV beams, respectively, compared to radiation without post incubation at 37 °C (cf. Figure 2a). The number of CHLS was more significant, contributing 1.8±0.4 and 4.8±0.9 times more DSBs/Gy/plasmid in the 100 kVp beam and 6 MV beams, respectively, compared to radiation without post incubation at 37 °C (cf. Figure 2b). This also implies that the 6 MV irradiation resulted in ~2.7 times greater CHLS damage than the 100 kVp irradiation. The increase in contribution of IOBD after enzymatic treatment with incubation was approximately the same for both beams, i.e., 1.83±0.04 and 1.79±0.01 times more SSBs/Gy/plasmid in the 100 kVp and 6 MV beams, respectively, compared to irradiation and incubation alone (cf. Figure 2a). Finally, COBD increased DSBs/Gy/plasmid by factors of 2.3±0.3 and 2.91±0.08 in the 100 kVp and 6 MV beams, respectively, compared to irradiation and incubation alone (cf. Figure 2b). In summary, these results show that the degree of increase in both CHLS and COBD damage were greater with the 6 MV irradiation, while the 100 kVp irradiation yielded a greater amount of DSB damage.

### 2.4. Number of DSBs per SSB and DDE

To directly compare the DDE of each beam, we first found the following ratios of the DNA damage parameters shown in Table 4: DSBs per SSB, CHLS per IHLS, and COBD per IOBD. Next, we computed the ratios of these values between 100 kVp and 6 MV beam, as displayed in Table 5. Table 4 and Table 5 clearly show that the 100 kVp X-ray beam causes an approximately 5-fold increase in DSBs per SSB, and a 20% increase in COBD per IOBD compared to the 6 MV X-ray beam (Table 5).

## 3. Discussion

In this study, we have demonstrated the feasibility of constructing a DNA damage detector that can be shipped thousands of kilometers for irradiation and shipped back to the site of origin for analysis. In addition, we have shown that the shipped detectors were sensitive enough to detect differences in the extent of DNA damage of 100 kVp and 6 MV X-ray beams, particularly as measured by the $DDE_{DSB/SSB}$ (Equation (6)). These DNA-based detectors quantify SSB and DSB ionizing radiation damage as conformational changes in supercoiled plasmid DNA to open circular and linear, respectively. We also incubated the irradiated samples at 37 °C to assess post irradiation heat labile sites, and separately incubated the samples at 37 °C with Nth and Fpg enzymes to quantify isolated and clustered oxidized base damage. While we found all categories of DNA damage, except for DSBs/Gy/plasmid, to be greater for the 6 MV beam versus the 100 kVp beam (Table 3), the severity of the damage as indicated by the $DDE_{DSB/SSB}$ and $DDE_{COBD/IOBD}$ was greater for the higher LET 100 kVp beam irradiation. Of the DDE indicators defined in this study (Equations (6)–(8)), $DDE_{DSB/SSB}$ yielded the greatest DDE ratio, approximately five-fold greater in the 100 kVp beam than the 6 MV beam (Table 5). Hence, despite the 6 MV and 100 kVp beams having mean LETs of ~0.2 and ~2.3 keV/μm, respectively, a difference of ~2.1 keV/μm, the $DDE_{DSB/SSB}$ provides a useful metric for comparing the two beam types in terms of their ability to cause potentially detrimental DNA damage (Table 6).

It should be noted that the free radical scavenging capacity of our DNA solution$\;\left(\sim 5.4\times 10^{5}{\;s}^{-1}\right)$ was ~555 times smaller than that of an average cell ($\sim 3\times 10^{8}{\;s}^{-1}).$ Under this low scavenging capacity, the main DNA damage observations for the 6 MV versus 100 kVp X-rays representing low versus higher LET X-ray radiations can be summarized as follows: (1) All types of DNA damage, except for DSBs, decrease with increased LET; (2) The ratios of DSBs per SSBs and COBD per IOBD increase with increased LET. The ratio of CHLS per IHLS may also increase, but the uncertainty in this metric was too large for this observation to be definitive.

The above observations are consistent with previous studies quantifying DNA damage under low free radical scavenging conditions for which indirect DNA damage increases relative to direct damage [20,23,24,26,27,28,29]. The increase in indirect DNA damage is due to increased free radical lifetime and its associated longer diffusion distance, which for low LET radiation leads to an increase in SSBs. However, for higher LET radiation there is a decrease in SSB DNA damage because the additional boost in free radical concentrations also increases the probability of local intra-track recombination [29]. Furthermore, the higher LET radiation will still induce DSBs via direct action, resulting in an increased DSB-to-SSB ratio.

The advantage of using low scavenging conditions for a DNA-based detector is the production of more DNA damage at lower radiation doses, which can be quantified more readily using agarose gel electrophoresis. This quantification allows for the comparison of the DNA damaging effectiveness of radiations having different LET via observation 2 above and Equations (6)–(8). Furthermore, studies investigating DNA damage as a function of scavenging conditions found that for photon radiation, the decrease in SSBs and DSBs is inversely proportional and linear to the increase in scavenging capacity, where the linear proportionality constant is dependent on both temperature and type of damage (SSB versus DSB) [20,26,28]. Hence, for a given LET radiation, the results acquired under the low scavenging conditions used in this study can be directly related to cell mimetic scavenging conditions.

It has been known for some time that higher LET radiation (up to ~300 keV/µm) [30] results in greater cell kill than lower LET radiation [31]. As DNA is thought to be the target of this cell kill, it appears counterintuitive that most types of DNA damage, including DSBs in some cases, are greater in the lower versus higher LET radiation, even under cell mimetic scavenging conditions. The explanation for this apparent contradiction is that there is less dense randomly distributed DNA damage that is more conducive to repair in low LET radiation, whereas high LET radiation possesses less overall damage that is more densely clustered, and hence more lethal [32]. In other words, the particle track structure of high LET radiation produces clustered DNA damage sites that are more difficult for cells to repair. Previous studies using conformational changes in supercoiled DNA and agarose gel electrophoresis for quantifying DNA damage have taken observation 2 above to support this explanation [24,25,26,28]. In further support of this, a study by Pang et al. used atomic force microscopy to resolve spatial distributions of radiation induced DSBs in plasmid DNA and provided direct evidence that particle track structure of high LET radiation plays a key role in producing more lethal DNA damage to cells [33]. Their study showed that the lower LET electron radiation induces a more uniform distribution of DSBs, whereas the higher LET neutron radiation induces a greater proportion of DSBs clustered in small spatial regions (up to 150 bp), i.e., DNA fragmentation. In addition, a recent computational study by Henthorn et al. [32], which simulated both pBR322 plasmid DNA and cell DNA damage, also supports the idea that cell kill is related to the spatial clustering of energy depositions. In light of the evidence from these other independent approaches, it is clear that the ratios given in observation 2, which can be viewed as surrogate measures for the degree of spatial clustering of DNA damage, can be used as a metric for the lethality of DNA damage of X-ray radiation with different LET. Furthermore, in this study, we have shown that these ratios can be used to compare the lethal DNA damaging ability of such radiations via the DDE definitions given by Equations (6)–(8); particularly $DDE_{DSB/SSB}$ as mentioned above.

With the rising use of proton and carbon ion radiotherapy beams, which can lead to radiation treatment plans that have spatially varying LET and corresponding spatial variations in biological effectiveness, it is important to have a tool to investigate such variations and provide quality control for patient-specific treatment plans. That is, phenomenologically-based models that use LET_D_ as a surrogate or intermediate quantity for the cumulative relative biological effectiveness (RBE) of spatially varying LET beams would benefit from independent quality control measurements such as $DDE_{DSB/SSB}$. Toward this end, this study demonstrates the feasibility of a transportable DNA-based dosimeter that can discern changes in LET at least as small as 0.5 keV/μm via the $DDE_{DSB/SSB}$ metric of radiochemical damage of plasmid DNA. Future work will apply these dosimeters to investigate the relationship between $DDE_{DSB/SSB}$ and LET_D_ in the spread-out Bragg peak of a proton beam and in patient-specific proton treatment plans with variable LET_D_.

## 4. Materials and Methods

### 4.1. DNA-Based Detector Design and Construction

**Figure 3:**
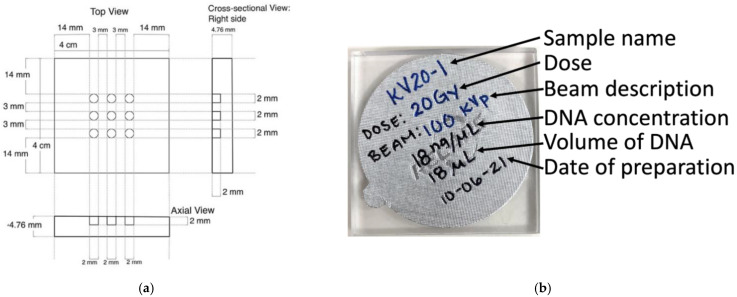
(**a**) Schematic of the DNA-based detector illustrating the position and size of the nine cavities that hold the DNA samples; (**b**) A sealed DNA-based detector ready for irradiation. Seal is covering wells containing DNA and has label information.

The phantoms used to encase the DNA were designed in San Diego and constructed in Toronto from nearly tissue equivalent polycarbonate (McMaster-Carr Supply Company, Elmhurst, IL, USA) ($\mathrm{density}=1.19\frac{g}{{\mathrm{cm}}^{3}})$. The dimensions of the phantom, with nine $6.3\;{\mathrm{mm}}^{3}\;$cylindrical cavities to encase the DNA samples, is shown in Figure 3a. We pipetted 3 μL of pBR322 plasmid DNA (Thermo Fisher Scientific, Inc., Waltham, MA, USA) solution with a DNA density of 18 ng/μL into each cavity. To encase the DNA samples into the phantom cavities, we applied an aluminum foil sticker seal of thickness $\left(3.50\pm 0.05\right)\times 10^{-2}\;\mathrm{mm}\;$(RECAPS, Foshan, China) on top of the phantom, with the sample name, radiation dose to be delivered, beam energy, DNA concentration, volume of DNA, and date of preparation written on the seal, as seen in Figure 3b. This encasement protects the DNA contents in the cavities from contamination and evaporation. To further protect the samples and prevent evaporation during shipment, we also placed the DNA-based detectors in individual vacuum sealed bags for shipment to Toronto. Finally, we packed the detectors on ice in a Styrofoam insulated box for overnight shipment from San Diego to Toronto for irradiation.

### 4.2. Plasmid pBR322 DNA Preparation and Recovery

We obtained pBR322 plasmid DNA at a concentration of 500 ng/μL at ~95% fraction of supercoiled conformation (Thermo Fisher Scientific, Inc.). This plasmid DNA is derived from E. coli bacterium. To maintain the supercoiled conformation, the DNA is stored at −20 °C in 10 mM Tris and 1 mM EDTA (i.e., TE buffer, Thermo Fisher Scientific, Inc.) at a pH of 7.6. The DNA is prepared for the detectors by diluting it down to a concertation of 18 ng/μL using 20 mM potassium phosphate (PP) to maintain the pH and reduce the free radical scavenging capacity.

A total of 14 detectors were prepared. Two control detectors received no dose, and the remaining detectors received radiation doses of 10, 20, and 30 Gy in duplicate from either a 100 kVp or a 6 MV X-ray beam. We stored one of the controls at −20 °C in San Diego while the other was shipped to Toronto with the samples to be irradiated where it was also stored at −20 °C. We used the controls to determine the impact that shipping and handling had on the DNA samples.

### 4.3. Shipment of DNA-Based Detectors

We wrapped the DNA-based detectors in absorbent paper towels and placed them in a Ziploc^®^ storage bag (SC Johnson, Inc., Racine, WI, USA). To slow degradation of the DNA conformations, we placed icepacks around the bag containing the detectors and used a Styrofoam shipping box for the overnight FedEx shipment both to Toronto and back to San Diego.

After the DNA-based detectors were shipped back to San Diego we removed the aluminum seals and pipetted the DNA out of the cavities and placed them into prelabeled microcentrifuge tubes. We diluted the samples to a concentration of 11.6 ng/μL using TE buffer. This concentration was determined as the concentration necessary for enzymatic treatment to maintain a treatment volume of 100 μL per sample [34,35].

### 4.4. Irradiation of Samples

Upon arrival in Toronto, the samples were stored at −20 °C to preserve conformational stability of the pBR322 DNA samples in the detector cavities until they could be irradiated. Before irradiation, we removed the DNA-based detectors from the freezer and left them to thaw at room temperature for approximately 11 min. After the DNA solution thawed, we removed from the vacuum seal bags, and irradiated them using the beam energy and dose as indicated on the detector labels.

**Figure 4:**
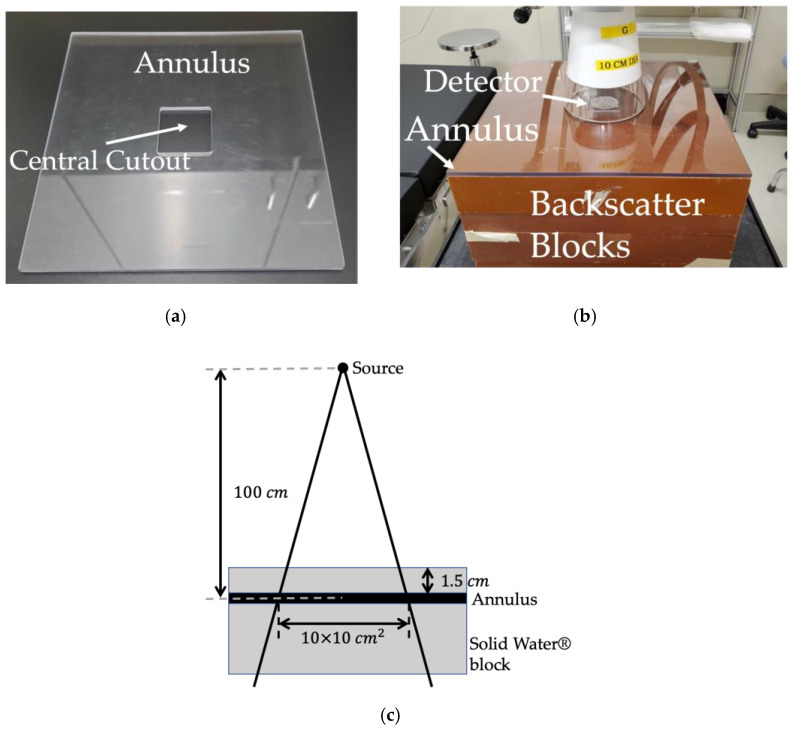
(**a**) Polycarbonate annulus with a central cutout for the insertion of the DNA-based detectors; (**b**) Picture of the setup for 100 kVp beam irradiations; (**c**) Schematic for 6 MV beam irradiations setup.

We used the Xstrahl 300 Orthovoltage unit (Xstrahl, Inc., Suwanee, GA, USA) with a 10 cm diameter cone insert to deliver the 100 kVp X-ray beam doses. The dose calibration for this system followed the AAPM Task Group 61 in-air protocol, which yields a total dose uncertainty of 3.5% [36]. Before delivering the radiation dose to the DNA-based detectors, we corrected for the attenuation by the aluminum seals that we used to encase the DNA in the detector cavity. We did this by dividing the radiation output measured without the aluminum seal by the radiation output measured with the aluminum seal using a parallel plate chamber (Exradin^®^ A11 Ion Chamber, Standard Imaging, Inc., Middleton, WI, USA). This gave us a correction factor of 1.027, and we multiplied the open beam radiation delivery calculations by this correction factor to ensure that the DNA sample sitting in the cavity under the seal received the total intended dose. For the irradiations, we inserted the DNA-based detectors at the center of a polycarbonate annulus we constructed (Figure 4b), which in turn was placed on top of 18 cm of Solid Water^®^ (Sun Nuclear Corporation, Melbourne, FL, USA), pictured in Figure 4b. We used the annulus to eliminate any air gap.

We delivered the 6 MV X-ray beam dose using the Elekta Synergy^®^ linear accelerator (Elekta, Inc., Stockholm, Sweden). The dose calibration for this system followed the AAPM Task Group 51 protocol, which yields a total dose uncertainty of 2.1% [37]. We used a 100 cm source-to-axis distance setup as shown in Figure 4c, with a $10\times 10{\;\mathrm{cm}}^{2}$ field size defined at isocenter. To account for buildup to maximum depth dose, we placed a 1.5 cm Solid Water^®^ block on top of the DNA-based detectors, which were inserted in the polycarbonate annulus (cf., Figure 4a,c). The attenuation effect of the aluminum seal on the 6 MV beam was measured by placing a parallel plate chamber (Exradin^®^ A11 Ion Chamber, Standard Imaging, Inc.) at a depth of 1.5 cm inside a solid water phantom (i.e., the depth at which the plasmid DNA in the detector cavity was also placed as shown in Figure 4c). Using this setup, the beam output was measured with and without the aluminum seal, and it was found to be the same. Hence, as expected, the presence of the thin aluminum seal did not alter the maximum depth dose for the 6 MV beam, and as such, we did not need to apply a correction factor for it.

The control DNA samples were not irradiated, but we left them out at room temperature for the same duration as the irradiated samples to control for any DNA conformational degradation that could occur due to freezing and thawing of the DNA samples.

### 4.5. Agarose Gel Electrophoresis

A portion of each DNA sample was prepared with additional TE buffer and loading dye for agarose gel electrophoresis (AGE). We prepared 0.9% agarose gels having 28 wells seeded with 3 ng of a DNA sample per well. We ran the gels for 1 h and 20 min at 100 mA and 80 V.

**Figure 5:**
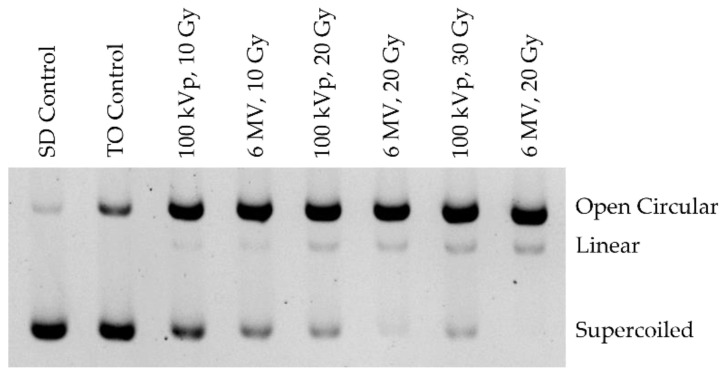
A representative gel run showing the three plasmid DNA conformations separated into bands. The DNA in this example was irradiated but had no enzymatic treatment applied. For easy visualization of bands, the image is shown over exposed and inverted.

Plasmid DNA is advantageous in studying ionizing radiation-induced DNA damage as it possesses three conformations: supercoiled, open circular, and linear, which correspond to no damage, SSB, and DSB damage, respectively. AGE separates the DNA sample into three distinct bands, corresponding to the three confirmations, as seen in Figure 5. The three DNA conformations travel at different velocities through the agarose gel matrix, with the supercoiled conformation traveling the fastest and furthest, followed by linear, and open circular (Figure 5).

Upon completion of the gel runs, we applied the ultra-sensitive SBYR Gold nucleic acid gel stain (Thermo Fisher Scientific, Inc.) to the gels for 1 h and 30 min. Once stained, we placed the gels on an UltraBright LED Transilluminator (MaestroGen, Inc., Hsinchu, Taiwan), which itself was in light-tight enclosure. We took images of the gels using a 14 bit-depth camera, ZWO ASI178MC CMOS (High Point Scientific, Inc., Montague, NJ, USA) at an optimal exposure to prevent pixel saturation. We also performed a calibration gel to ensure that the band intensity signals were linearly proportional to DNA mass up to the DNA mass seeded in this study. We ran 4 gels, each with 28 lanes, to obtain 14 intensity points per sample group (i.e., SDSU control, Shipped Control, 100 kVp 10 Gy, 6 MV 10 Gy, 100 kVp 20 Gy, 6 MV 20 Gy, 100 kVp 30 Gy, and 6 MV 30 Gy). Images and band intensities of the agarose gel electrophoresis data are given in the Supplementary Files.

### 4.6. Percent Yields and Curve Fitting

We used the Core Laboratory Image Quantification Software (CLIQS) (TotalLab Ltd., Newcastle upon Tyne, UK) to find the DNA conformational band intensities from the gel images. That is, the sum of all the pixel intensities of a given band constituted the total intensity of the band. To find the fractional yield of each DNA conformation from a given lane, we divided the total intensity of each DNA band by the sum of the three bands in the lane. We found the mean standard deviation over the 14 samples of each type of DNA conformation yield. We plotted these average percent yields of supercoiled, linear, and open circular conformations as a function of dose, for each beam energy, using the robust curve-fitting procedure presented by McMahon and Currell [25] using the following equations:

(1) $$S=S_{0}e^{-\left(\beta_{s}+\beta_{D}\right)D}$$

(2) $$C=e^{-\beta_{D}D}(C_{0}e^{-\frac{1}{2}\beta_{s}^{2}\rho D^{2}}+S_{0}(e^{-\frac{1}{2}\beta_{s}^{2}\rho D^{2}}-e^{-\beta_{s}D}))$$

(3) $$L=1-\left(C_{0}+S_{0}\right)e^{-\left(\beta_{D}D+\frac{1}{2}\beta_{s}^{2}\rho D^{2}\right)}$$

where D is the radiation dose in Gy and ρ is probability of two SSBs occurring within 10 base pairs to become a DSB. C, L, and S  represent the percent yield of the open circular, linear, and supercoiled plasmid DNA conformations, respectively. $C_{0}$, $L_{0}$, and $S_{0}$ are the initial percent yield values of the open circular, linear, and supercoiled conformation, respectively, given by the control samples. The probability parameters $\beta_{S}$ and $\beta_{D}$ represent the rate SSBs and DSBs occur per Gy per plasmid. We computed these parameters using the evolutionary nonlinear solver in Microsoft Excel (Microsoft, Inc., Redmond, WA, USA) to minimize:

(4) $$\chi^{2}=\frac{1}{3}\left(\left(\chi_{S}^{2}+\chi_{C}^{2}\right)+\left(\chi_{S}^{2}+\chi_{L}^{2}\right)+\left(\chi_{C}^{2}+\chi_{L}^{2}\right)\right)=\frac{2}{3}\left(\chi_{S}^{2}+\chi_{C}^{2}+\chi_{L}^{2}\right),$$

where

(5) $$\chi_{\gamma }^{2}=\overset{N-1}{\underset{i=0}{\sum }}{\left(\frac{y_{i}-y\left(D_{i};\beta_{S},\beta_{D}\right)}{\sigma_{i}}\right)}^{2}$$

In Equation (5), $y_{i}$ is the mean measured yield at dose $D_{i}$, $\sigma_{i}$ is the standard deviation of the data group being evaluated, γ is plasmid DNA isoform S, C, or L, and $y\left(D_{i};\beta_{S},\beta_{D}\right)$ are the model functions, Equations (1)–(3), that are being fitted to the measurements. To find the standard error in the beta parameters, we utilized the asymptotic covariance matrix constructed from the product of the error variance of the model fit and the inverse of the Jacobian transpose multiplied by the Jacobian of Equations (1)–(3) with respect to the $\beta_{S}$ and $\beta_{D}$ parameters at the measured dose points. We multiplied this standard error by the t-distribution value corresponding to the 68% confidence interval.

### 4.7. Enzyme Digest and Incubation

To quantify isolated and clustered oxidized base damage, we applied an enzymatic treatment with Fpg (formamimopyrimidine DNA glycosylase) (New England BioLabs, Inc., Ipswich, MA, USA) and Nth (endonuclease III) (New England BioLabs, Inc.) enzymes to the DNA samples. This treatment exposes the isolated and clustered oxidized base damage as additional SSBs and DSBs. We adapted the enzyme digest procedure from New England BioLabs procedures, so that both enzymes were used simultaneously in a double digest. A total 10 μL double digest solution contained 0.1 μg of a DNA sample, 0.1 μL of NEBuffer1 (New England BioLabs, Inc.), 0.81 μL of S-adenosyl-methionine (SAM) (New England BioLabs, Inc.), 0.09 μL of Bovine Serum Albumin (BSA) (New England BioLabs, Inc.), and 0.2 μL of each enzyme [34,35]. We incubated the double digest solution at 37 °C for 60 min. After enzymatic treatment, we performed AGE to quantify the total SSBs and DSBs, and we found the percent yields of each conformation and the $\beta_{S\left(enz\right)}$ and $\beta_{D\left(enz\right)}$ parameters as described in Section 4.6 for the nontreated DNA samples.

To account for and quantify any additional DNA degradation, specifically radiation-induced heat-labile sites, exposed by the incubation during the enzymatic treatment, the samples underwent incubation alone for the equivalent time (60 min) as the enzymatically treated samples. We computed the percent yields of each conformation and the parameters $\beta_{S\left(inc\right)}$ and $\beta_{D\left(inc\right)}$ as described in Section 4.6 for the nontreated DNA samples.

### 4.8. Characterization of the DNA Damage Types

We used the calculated $\beta_{S}$, $\beta_{D}$, $\beta_{S\left(enz\right)}$, $\beta_{D\left(enz\right)}$, $\beta_{S\left(inc\right)}$ and $\beta_{D\left(inc\right)}$ probability parameters to categorize and quantify the type of DNA damage induced to the DNA, as shown in Table 6. Before enzymatic treatment and incubation, the SSBs and DSBs quantified corresponded to prompt strand breaks induced by the ionizing radiation [38]. Incubation without enzymatic treatment corresponds to SSB and DSB yields that include IHLS and CHLS, respectively. Enzymatic treatment with incubation corresponds to SSB and DSB yields that included IOBD and COBD, respectively [22]. Hence, the types of corresponding damage were computed as shown in Table 3. All of the DNA damage yields are measured per Gy per plasmid.

### 4.9. DNA Damaging Effectiveness Metric

In the same spirit of the RBE definition for assessing the relative biological effectiveness of different types/energies of ionizing radiations on cells, we defined the following metrics to assess the DNA damaging effectiveness (DDE) of different types/energies of radiations on plasmid DNA:

(6) $$DDE_{DSB/SSB}={\left(\frac{DSB}{SSB}\right)}_{\mathrm{Test}}/{\left(\frac{DSB}{SSB}\right)}_{\mathrm{Ref}},\;$$

(7) $$DDE_{CHLS/IHLS}={\left(\frac{CHLS}{IHLS}\right)}_{\mathrm{Test}}/{\left(\frac{CHLS}{IHLS}\right)}_{\mathrm{Ref}},$$

(8) $$DDE_{CHLS/IHLS}={\left(\frac{COBD}{IOBD}\right)}_{\mathrm{Test}}/{\left(\frac{COBD}{IOBD}\right)}_{\mathrm{Ref}},$$

where the types of DNA damage are defined in Table 6 and the “Test” radiation (100 kVp X-ray beam in this study) is the radiation compared to a reference (“Ref”) radiation (6 MV X-ray beam in this study).

## Figures and Tables

**Table 1:** DNA yields for the control DNA kept in San Diego (SD) and the control DNA sent to Toronto (TO). DNA incubated at 37 °C for 1 h before agarose gel electrophoresis analysis is denoted by the subscript “inc”, and DNA incubated with enzymatic (Fpg and Nth) treatment at 37 °C for 1 h is denoted by the subscript “inc + enz”). Uncertainties represent the sample standard deviation.

Non-Irradiated Controls	Supercoiled (S)	Open Circular (OC)	Linear (L)
SD	96±1	4±1	0
TO	85±2	15±2	0
SD_inc_	94.5±0.4	5.2±0.4	0.3±0.2
TO_inc_	77.5±0.8	22.0±0.8	0.5±0.2
SD_inc+enz_	73±2	26±2	0.6±0.2
TO_inc+enz_	49±2	50±2	0.6±0.2

**Table 2:** Percentage of DNA damage computed from the yields in Table 1. Uncertainties represent the sample standard deviation.

Non-Irradiated Controls	% No Damage	% SSB (OC)	% DSB (L)	% IHLS (OC_inc_ − OC)	% CHLS (L_inc_ − L)	% IOBD (OC_inc+enz_ − OC_inc_)	% COBD (L_inc+enz_ − L_inc_)
SD	73±2	4±1	0	1±1	0.3±0.2	21±2	0.3±0.3
TO	49±2	15±2	0	7±2	0.5±0.2	28±2	0.1±0.3

**Table 3:** Types of DNA damage and corresponding probability parameters. Uncertainties represent 68% confidence intervals.

Types of Damage	100 kVp Beam	6 MV Beam
SSBs/Gy/plasmid $\left(\beta_{S\;}\right)$	$\left(9.4\pm 0.1\right)\times 10^{-2}$	$\left(1.581\pm 0.009\right)\times 10^{-1}$
IHLS/Gy/plasmid $\left(\beta_{S\;\left(\mathrm{inc}\right)}-\beta_{S\;}\right)$	$\left(3\pm 2\right)\times 10^{-3}$	$\left(8\pm 1\right)\times 10^{-3}$
IOBD/Gy/plasmid $\left(\beta_{S\;\left(\mathrm{enz}\right)}-\beta_{S\left(\mathrm{inc}\right)\;}\right)$	$\left(8.0\pm 0.3\right)\times 10^{-2}$	$\left(1.32\pm 0.01\right)\times 10^{-1}$
DSBs/Gy/plasmid $\left(\beta_{D}\right)$	$\left(7\pm 1\right)\times 10^{-4}$	$\left(2.4\pm 0.5\right)\times 10^{-4}$
CHLS/Gy/plasmid $\left(\beta_{D\;\left(\mathrm{inc}\right)}-\beta_{D\;}\right)$	$\left(5\pm 2\right)\times 10^{-4}$	$\left(9.0\pm 0.5\right)\times 10^{-4}$
COBD/Gy/plasmid $\left(\beta_{D\;\left(\mathrm{enz}\right)}-\beta_{D\left(\mathrm{inc}\right)\;}\right)$	$\left(1.6\pm 0.2\right)\times 10^{-3}$	$\left(2.19\pm 0.04\right)\times 10^{-3}$

Abbreviations: Probability parameters representing the amount of DNA damage per Gy per plasmid for samples post irradiation (β), post irradiation and incubation with no enzymatic treatment at 37 °C for 1 h $\left(\beta_{\;\left(\mathrm{inc}\right)}\right),$ and post irradiation and enzymatic treatment under incubation at $37\;{}^{\circ}C\;(\beta_{\;\left(\mathrm{enz}\right)}$).

**Table 4:** Summary of the ratios of DSB/SSB, CHLS/IHLS, and COBD/IOBD.

Ratio of Beta Parameter Values			
Beam Energy	No Incubation & No Enzyme DSB/SSB $\left(\times 10^{-3}\right)$	Incubated CHLS/IHLS $\left(\times 10^{-1}\right)$	Incubated & Enzyme COBD/IOBD $\left(\times 10^{-2}\right)$
100 kVp	7±2	2±2	2.0±0.2
6 MV	1.5±0.3	1.1±0.2	1.66±0.03

**Table 5:** The DNA damaging effectiveness (DDE) of the 100 kVp versus 6 MV X-ray beam.

DDE Ratios for 100 kVp (Test) Beam as Defined in Equations (6)–(8)		
$DDE_{DSB/SSB}$	$DDE_{CHLS/IHLS}$	$DDE_{COBD/IOBD}$
5±2	2±2	1.2±0.1

**Table 6:** Types of DNA Damage calculated from the probability parameters.

Type of DNA Damage	Probability Parameters (DNA Damage/Gy/Plasmid)
SSB	$\beta_{S}$
IHLS	$\beta_{S\;\left(inc\right)}-\beta_{S}$
IOBD	$\beta_{S\;\left(enz\right)}-\beta_{S\;\left(inc\right)}$
DSB	$\beta_{D}$
CHLS	$\beta_{D\;\left(inc\right)}-\beta_{D}$
COBD	$\beta_{D\;\left(enz\right)}-\beta_{D\;\left(inc\right)}$

Abbreviations: $\beta_{S}\;$ and $\beta_{D}$ = the probability parameters for the yields of SSBs/Gy/plasmid and DSBs/Gy/plasmid, respectively. Subscripts “(*inc*)” and “(*enz*)” represent the probability parameters for the yields of SSBs/Gy/plasmid and DSBs/Gy/plasmid for samples post incubation alone and post incubation with Nth and Fpg enzymatic treatment, respectively.

## Data Availability

Image and gel band intensity data files supporting the reported results can be found in the Supplementary Files.

## Supplementary Materials

The following supporting information can be downloaded at: https://www.mdpi.com/article/10.3390/ijms232012459/s1.
