# Supplementary material for: Assessing the DNA Damaging Effectiveness of Ionizing Radiation Using Plasmid DNA
Source: Int J Mol Sci. 2022 Oct 18;23(20):12459. doi: 10.3390/ijms232012459 (PMC9604049; doi:10.3390/ijms232012459)
Supplement: Supplementary file 1 [file ijms-23-12459-s001.zip › Agarose Gel Electrophoresis Images.pdf]

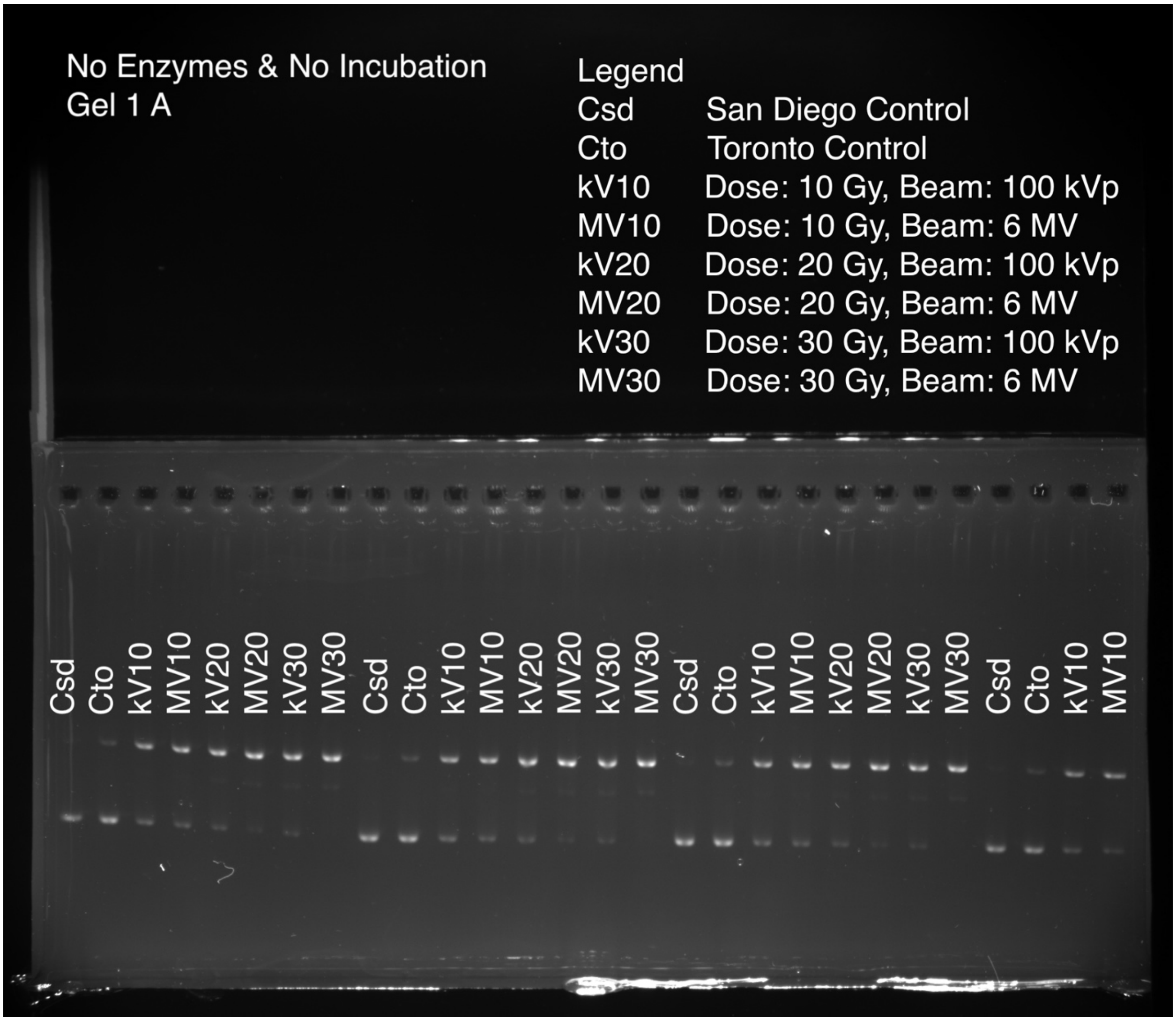
No Enzymes & No Incubation
Gel 1 A
Legend
Csd San Diego Control
Cto Toronto Control
kV10 Dose: 10 Gy, Beam: 100 kVp
MV10 Dose: 10 Gy, Beam: 6 MV
kV20 Dose: 20 Gy, Beam: 100 kVp
MV20 Dose: 20 Gy, Beam: 6 MV
kV30 Dose: 30 Gy, Beam: 100 kVp
MV30 Dose: 30 Gy, Beam: 6 MV
Csd Cto kV10 MV10 kV20 MV20 kV30 MV30 Csd Cto kV10 MV10 kV20 MV20 kV30 MV30 Csd Cto kV10 MV10 kV20 MV20 kV30 MV30 Csd Cto kV10 MV10

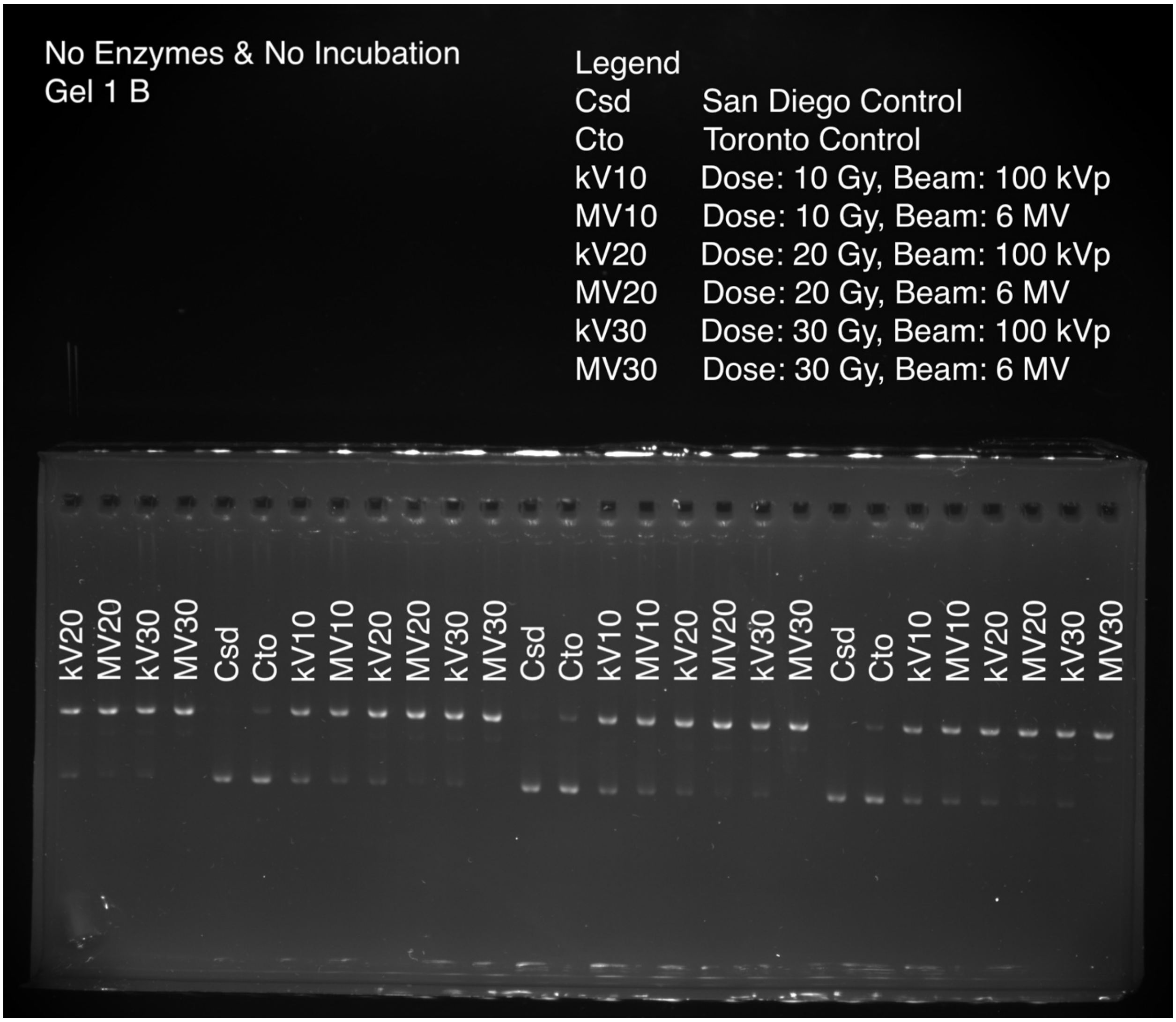
No Enzymes & No Incubation
Gel 1 B
Legend
Csd San Diego Control
Cto Toronto Control
kV10 Dose: 10 Gy, Beam: 100 kVp
MV10 Dose: 10 Gy, Beam: 6 MV
kV20 Dose: 20 Gy, Beam: 100 kVp
MV20 Dose: 20 Gy, Beam: 6 MV
kV30 Dose: 30 Gy, Beam: 100 kVp
MV30 Dose: 30 Gy, Beam: 6 MV
kV20 MV20 kV30 MV30 Csd Cto kV10 MV10 kV20 MV20 kV30 MV30 Csd Cto kV10 MV10 kV20 MV20 kV30 MV30 Csd Cto kV10 MV10 kV20 MV20 kV30 MV30

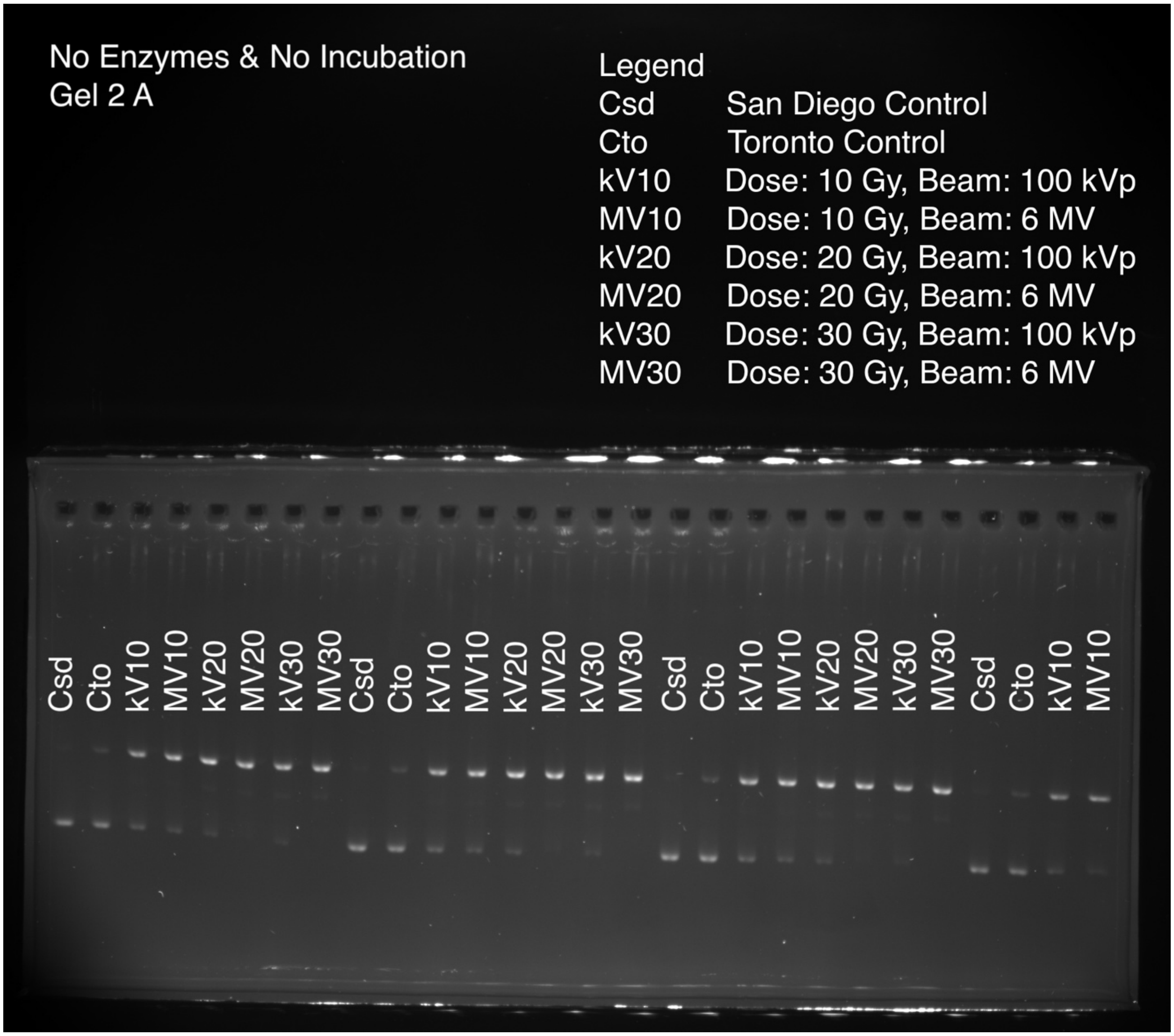
No Enzymes & No Incubation
Gel 2 A
Legend
Csd San Diego Control
Cto Toronto Control
kV10 Dose: 10 Gy, Beam: 100 kVp
MV10 Dose: 10 Gy, Beam: 6 MV
kV20 Dose: 20 Gy, Beam: 100 kVp
MV20 Dose: 20 Gy, Beam: 6 MV
kV30 Dose: 30 Gy, Beam: 100 kVp
MV30 Dose: 30 Gy, Beam: 6 MV
Csd Cto kV10 MV10 kV20 MV20 kV30 MV30 Csd Cto kV10 MV10 kV20 MV20 kV30 MV30 Csd Cto kV10 MV10 kV20 MV20 kV30 MV30 Csd Cto kV10 MV10

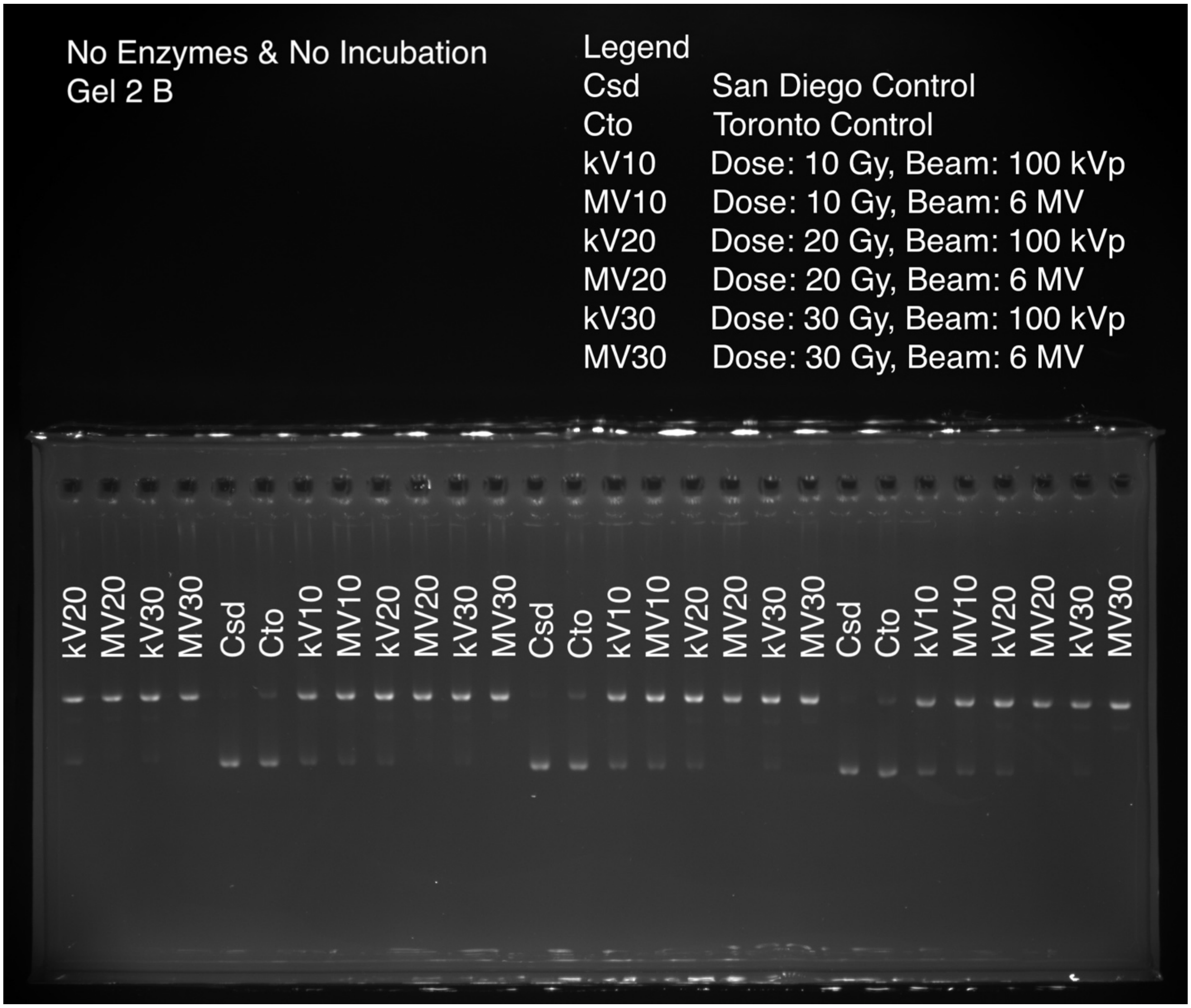
No Enzymes & No Incubation
Gel 2 B
Legend
Csd San Diego Control
Cto Toronto Control
kV10 Dose: 10 Gy, Beam: 100 kVp
MV10 Dose: 10 Gy, Beam: 6 MV
kV20 Dose: 20 Gy, Beam: 100 kVp
MV20 Dose: 20 Gy, Beam: 6 MV
kV30 Dose: 30 Gy, Beam: 100 kVp
MV30 Dose: 30 Gy, Beam: 6 MV
kV20 MV20 kV30 MV30 Csd Cto kV10 MV10 kV20 MV20 kV30 MV30 Csd Cto kV10 MV10 kV20 MV20 kV30 MV30 Csd Cto kV10 MV10 kV20 MV20 kV30 MV30

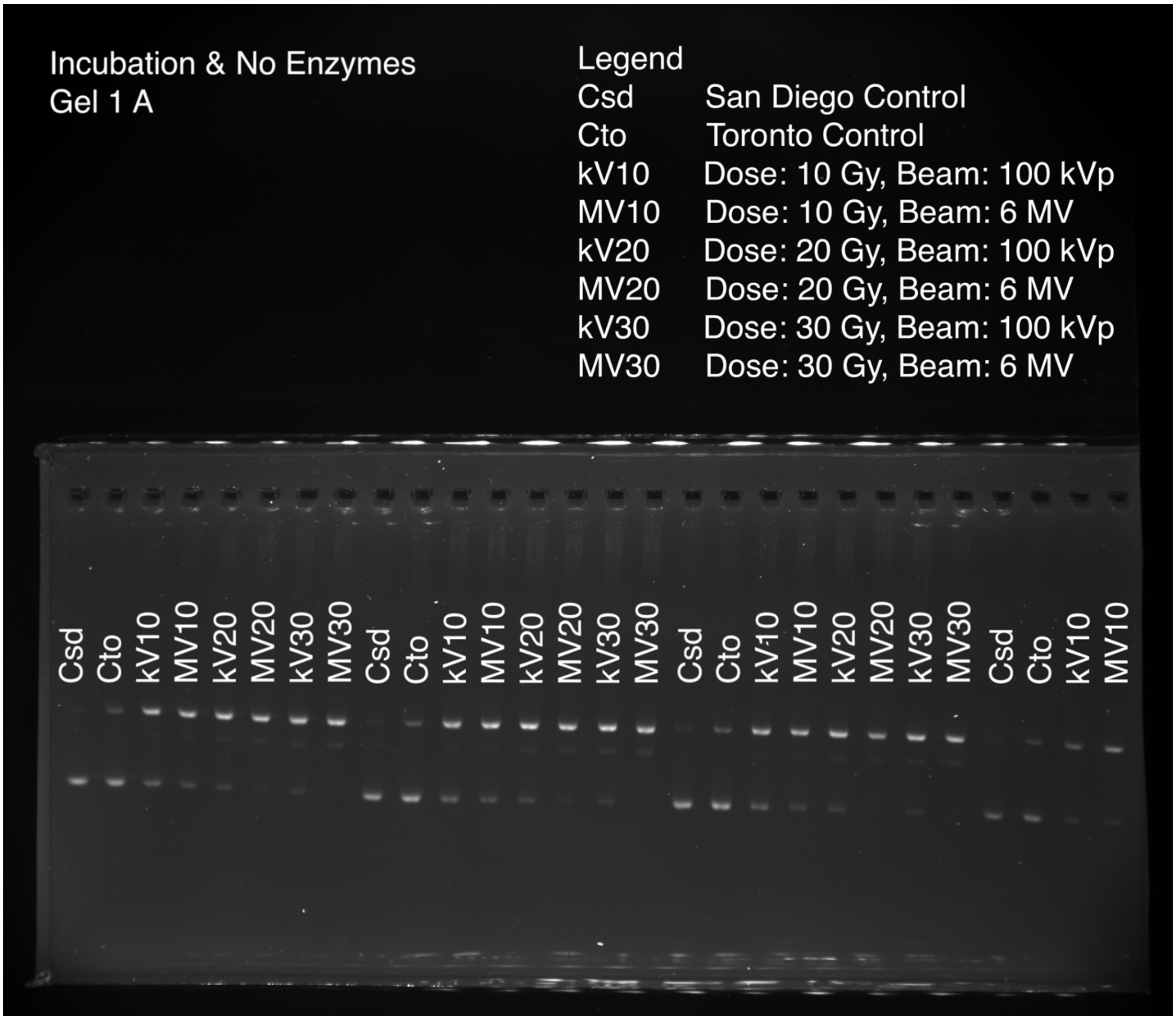
Incubation & No Enzymes
Gel 1 A
Legend
Csd San Diego Control
Cto Toronto Control
kV10 Dose: 10 Gy, Beam: 100 kVp
MV10 Dose: 10 Gy, Beam: 6 MV
kV20 Dose: 20 Gy, Beam: 100 kVp
MV20 Dose: 20 Gy, Beam: 6 MV
kV30 Dose: 30 Gy, Beam: 100 kVp
MV30 Dose: 30 Gy, Beam: 6 MV
Csd Cto kV10 MV10 kV20 MV20 kV30 MV30 Csd Cto kV10 MV10 kV20 MV20 kV30 MV30 Csd Cto kV10 MV10 kV20 MV20 kV30 MV30 Csd Cto kV10 MV10

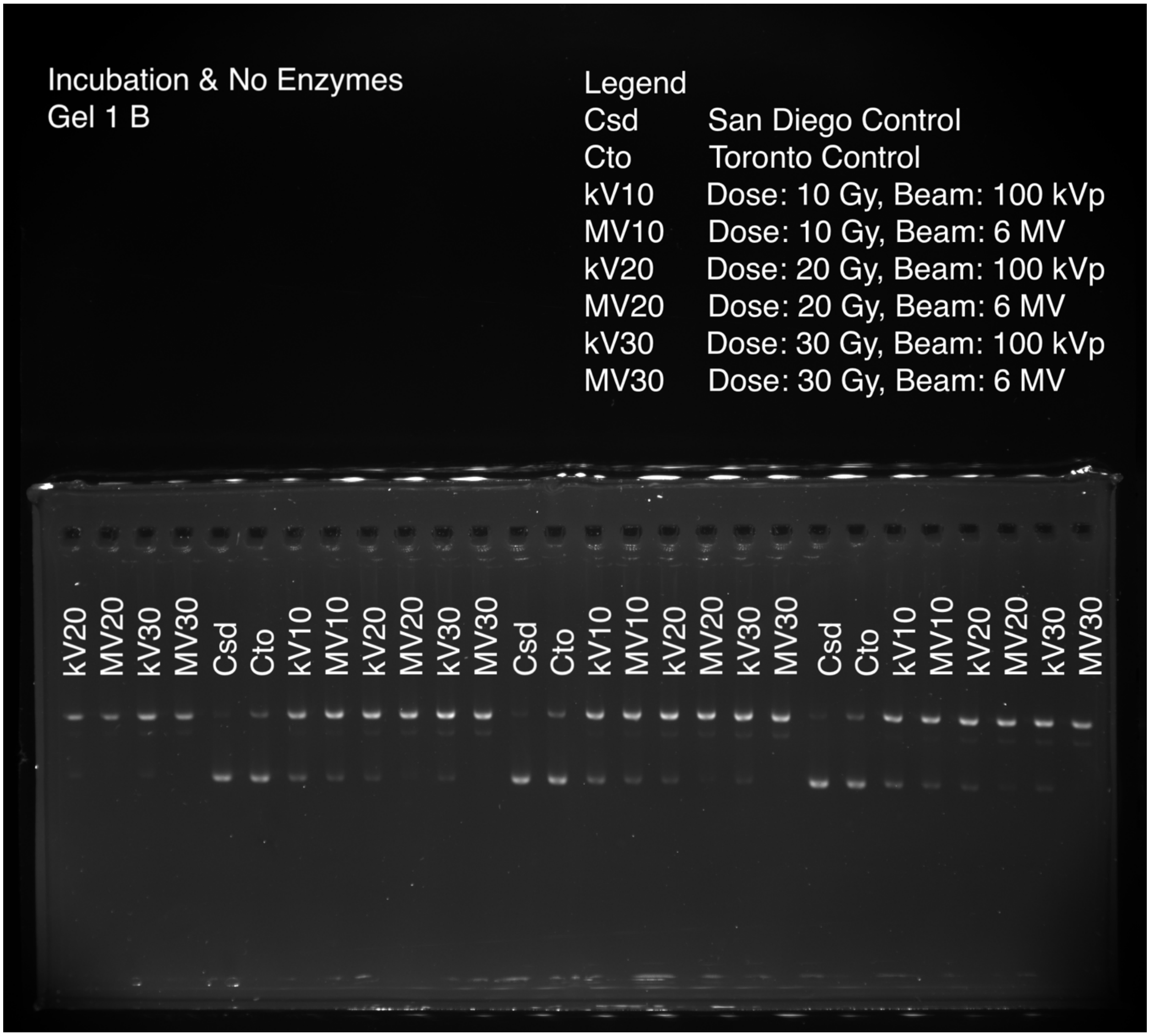
Incubation & No Enzymes
Gel 1 B
Legend
Csd San Diego Control
Cto Toronto Control
kV10 Dose: 10 Gy, Beam: 100 kVp
MV10 Dose: 10 Gy, Beam: 6 MV
kV20 Dose: 20 Gy, Beam: 100 kVp
MV20 Dose: 20 Gy, Beam: 6 MV
kV30 Dose: 30 Gy, Beam: 100 kVp
MV30 Dose: 30 Gy, Beam: 6 MV
kV20 MV20 kV30 MV30 Csd Cto kV10 MV10 kV20 MV20 kV30 MV30 Csd Cto kV10 MV10 kV20 MV20 kV30 MV30 Csd Cto kV10 MV10 kV20 MV20 kV30 MV30

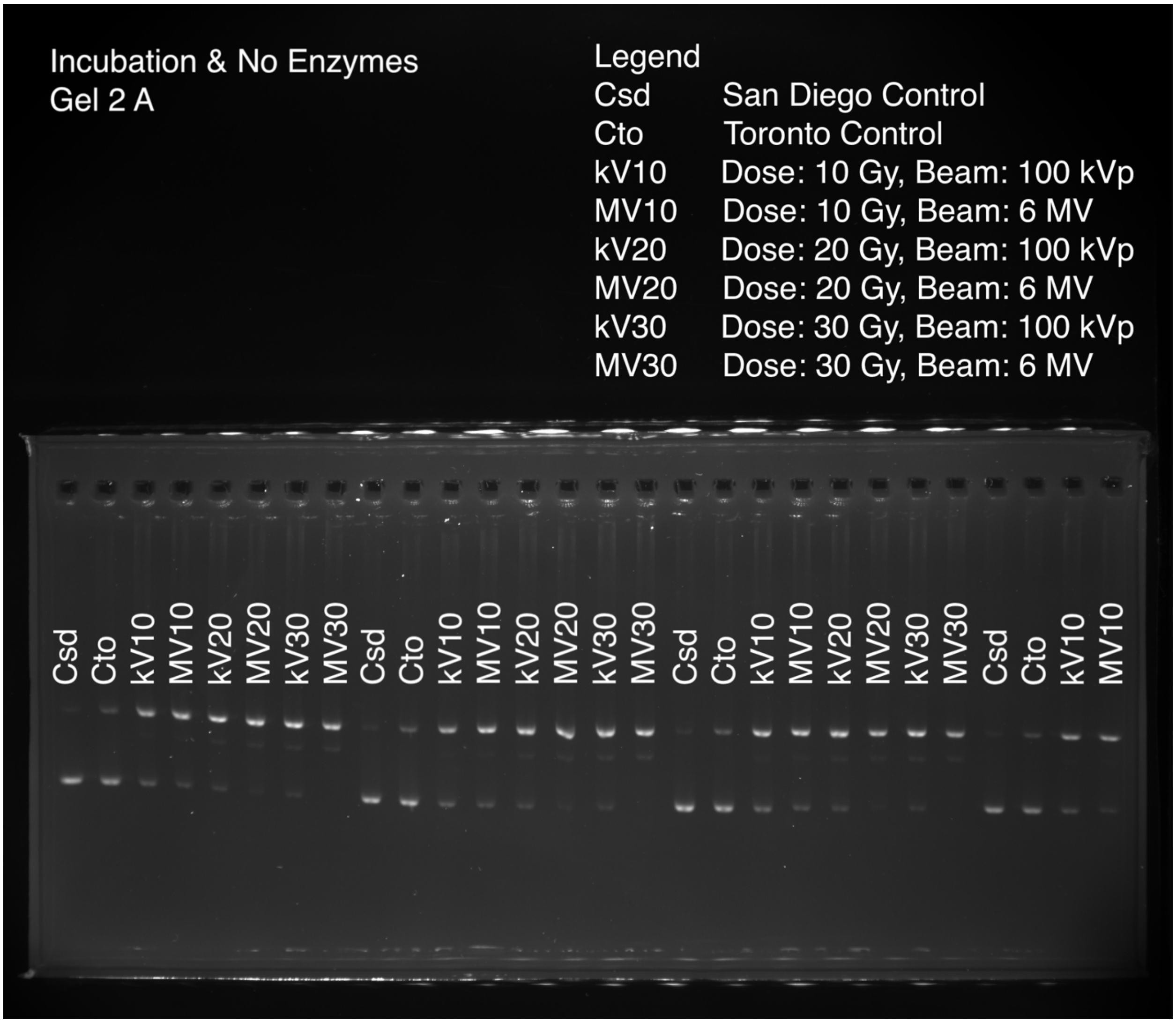
Incubation & No Enzymes
Gel 2 A
Legend
Csd San Diego Control
Cto Toronto Control
kV10 Dose: 10 Gy, Beam: 100 kVp
MV10 Dose: 10 Gy, Beam: 6 MV
kV20 Dose: 20 Gy, Beam: 100 kVp
MV20 Dose: 20 Gy, Beam: 6 MV
kV30 Dose: 30 Gy, Beam: 100 kVp
MV30 Dose: 30 Gy, Beam: 6 MV
Csd Cto kV10 MV10 kV20 MV20 kV30 MV30 Csd Cto kV10 MV10 kV20 MV20 kV30 MV30 Csd Cto kV10 MV10 kV20 MV20 kV30 MV30 Csd Cto kV10 MV10

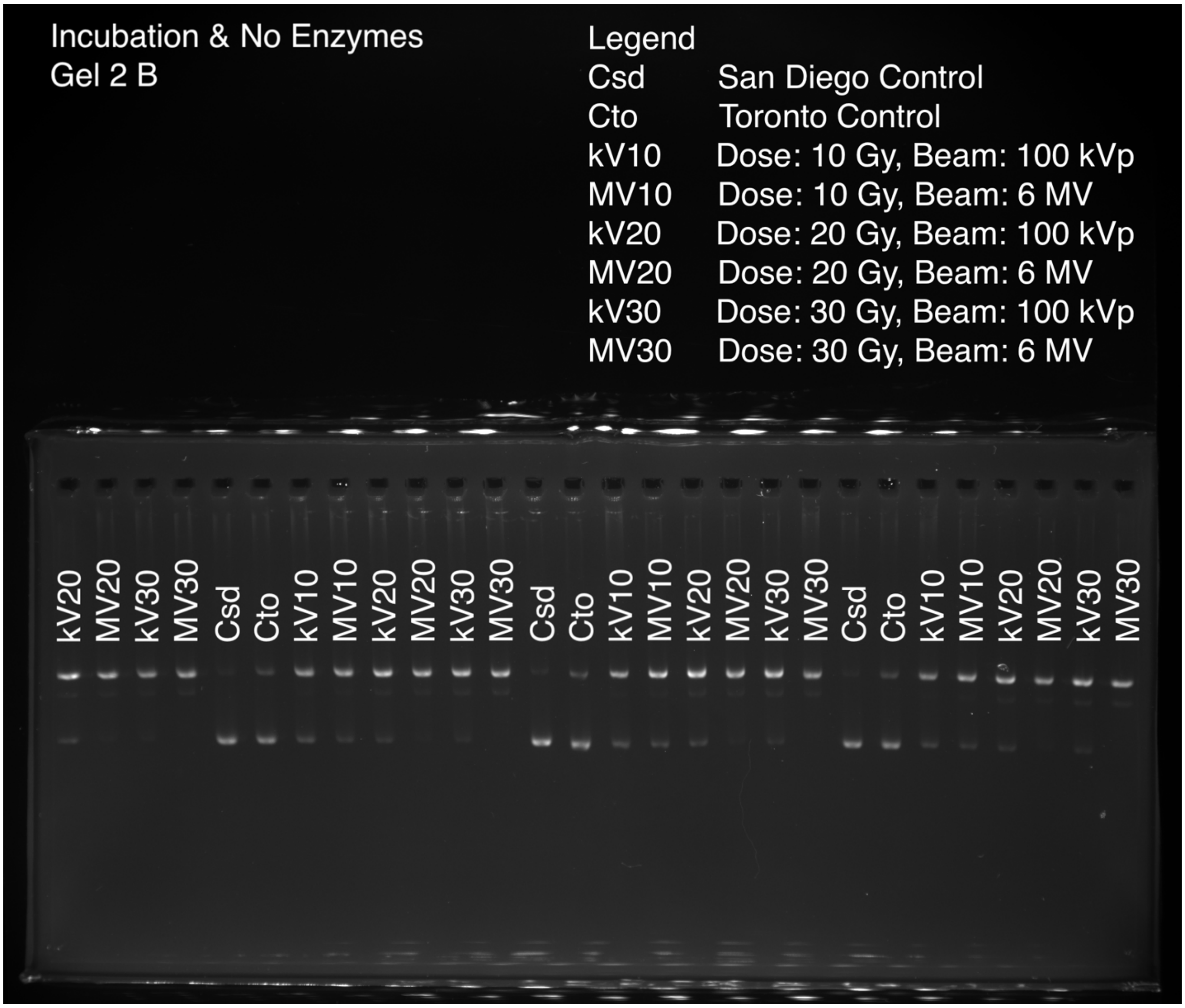
Incubation & No Enzymes
Gel 2 B
Legend
Csd San Diego Control
Cto Toronto Control
kV10 Dose: 10 Gy, Beam: 100 kVp
MV10 Dose: 10 Gy, Beam: 6 MV
kV20 Dose: 20 Gy, Beam: 100 kVp
MV20 Dose: 20 Gy, Beam: 6 MV
kV30 Dose: 30 Gy, Beam: 100 kVp
MV30 Dose: 30 Gy, Beam: 6 MV
kV20 MV20 kV30 MV30 Csd Cto kV10 MV10 kV20 MV20 kV30 MV30 Csd Cto kV10 MV10 kV20 MV20 kV30 MV30 Csd Cto kV10 MV10 kV20 MV20 kV30 MV30

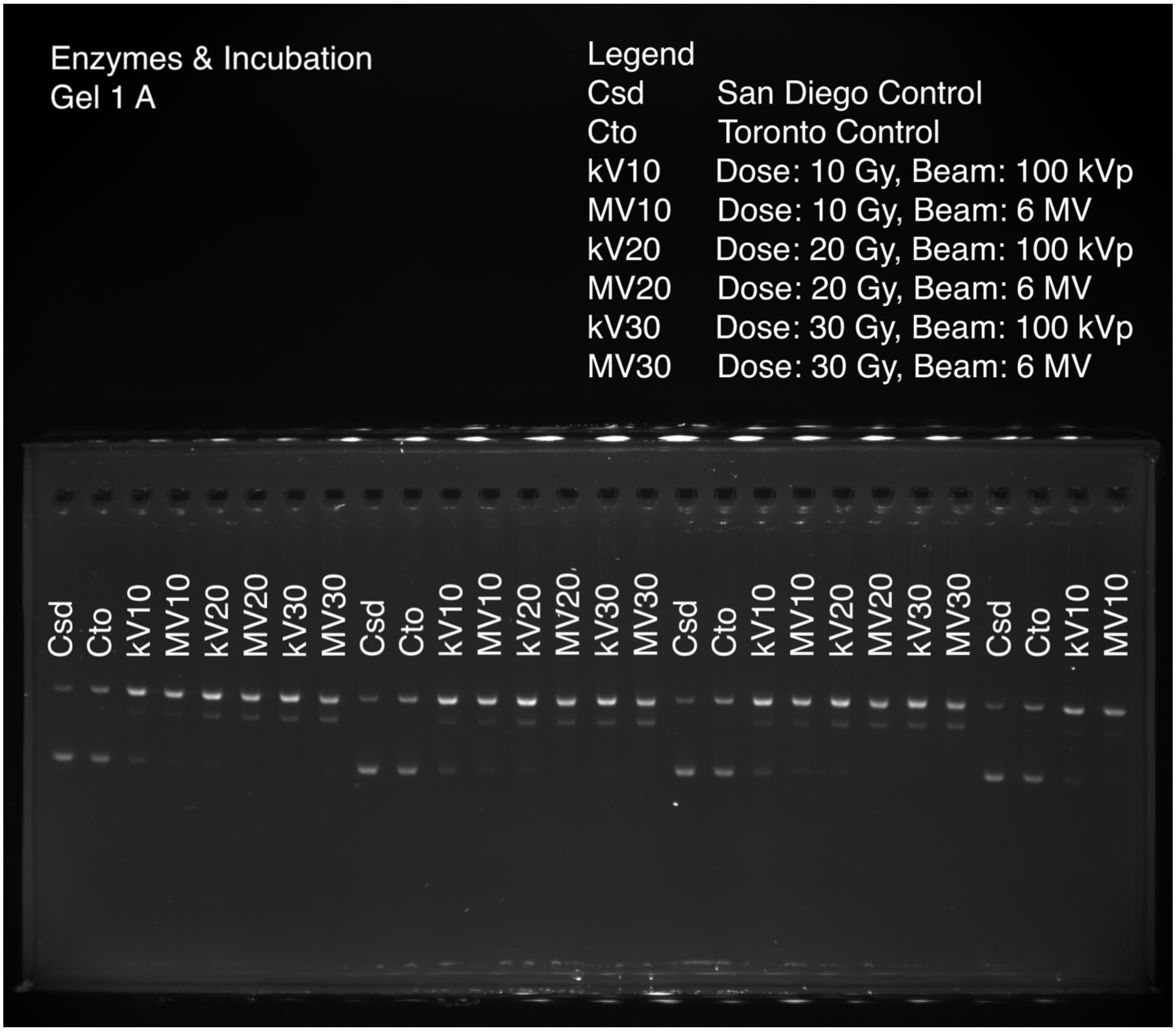
Enzymes & Incubation
Gel 1 A
Legend
Csd San Diego Control
Cto Toronto Control
kV10 Dose: 10 Gy, Beam: 100 kVp
MV10 Dose: 10 Gy, Beam: 6 MV
kV20 Dose: 20 Gy, Beam: 100 kVp
MV20 Dose: 20 Gy, Beam: 6 MV
kV30 Dose: 30 Gy, Beam: 100 kVp
MV30 Dose: 30 Gy, Beam: 6 MV
Csd Cto kV10 MV10 kV20 MV20 kV30 MV30 Csd Cto kV10 MV10 kV20 MV20 kV30 MV30 Csd Cto kV10 MV10 kV20 MV20 kV30 MV30 Csd Cto kV10 MV10

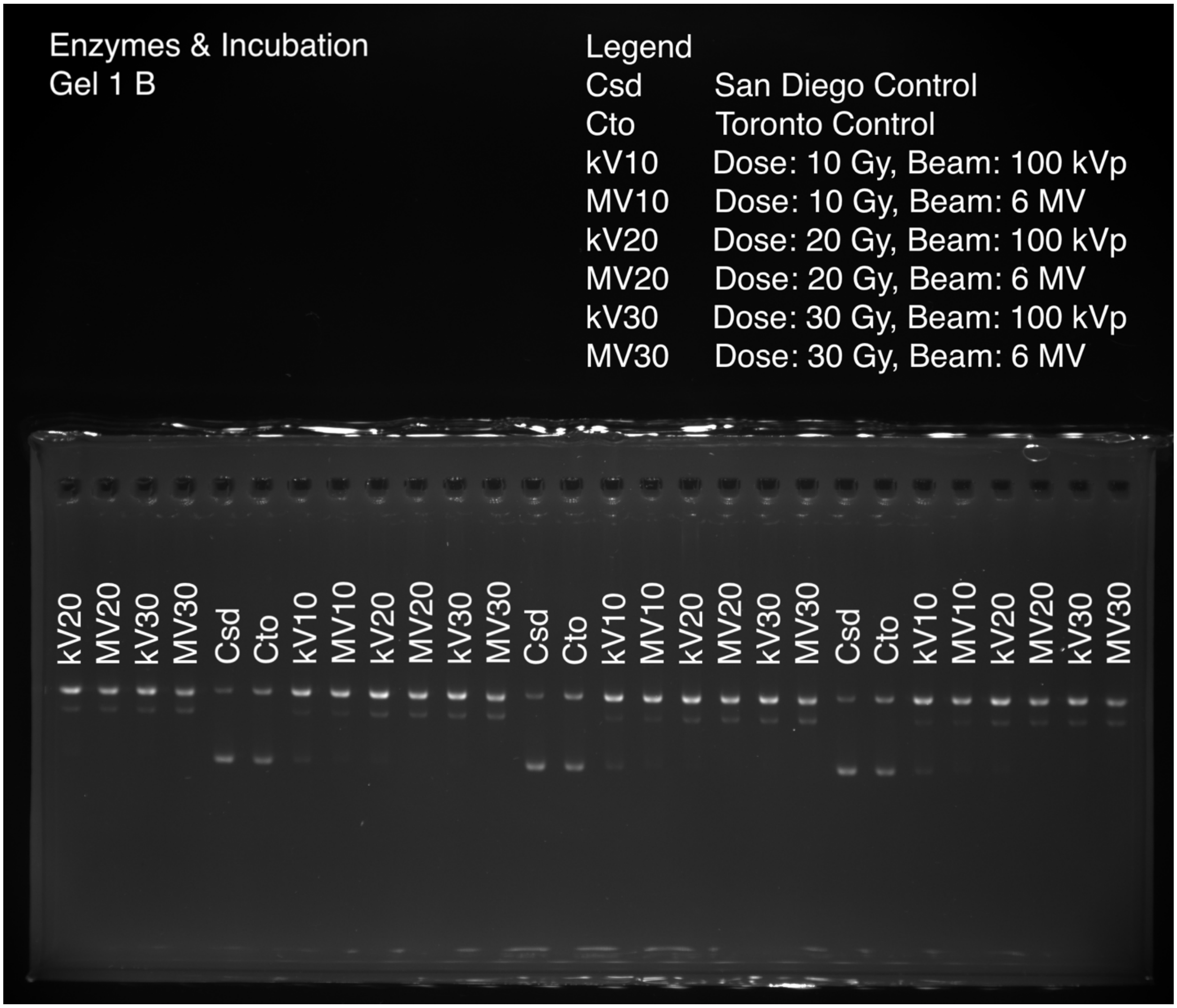
Enzymes & Incubation
Gel 1 B
Legend
Csd San Diego Control
Cto Toronto Control
kV10 Dose: 10 Gy, Beam: 100 kVp
MV10 Dose: 10 Gy, Beam: 6 MV
kV20 Dose: 20 Gy, Beam: 100 kVp
MV20 Dose: 20 Gy, Beam: 6 MV
kV30 Dose: 30 Gy, Beam: 100 kVp
MV30 Dose: 30 Gy, Beam: 6 MV
kV20 MV20 kV30 MV30 Csd Cto kV10 MV10 kV20 MV20 kV30 MV30 Csd Cto kV10 MV10 kV20 MV20 kV30 MV30 Csd Cto kV10 MV10 kV20 MV20 kV30 MV30

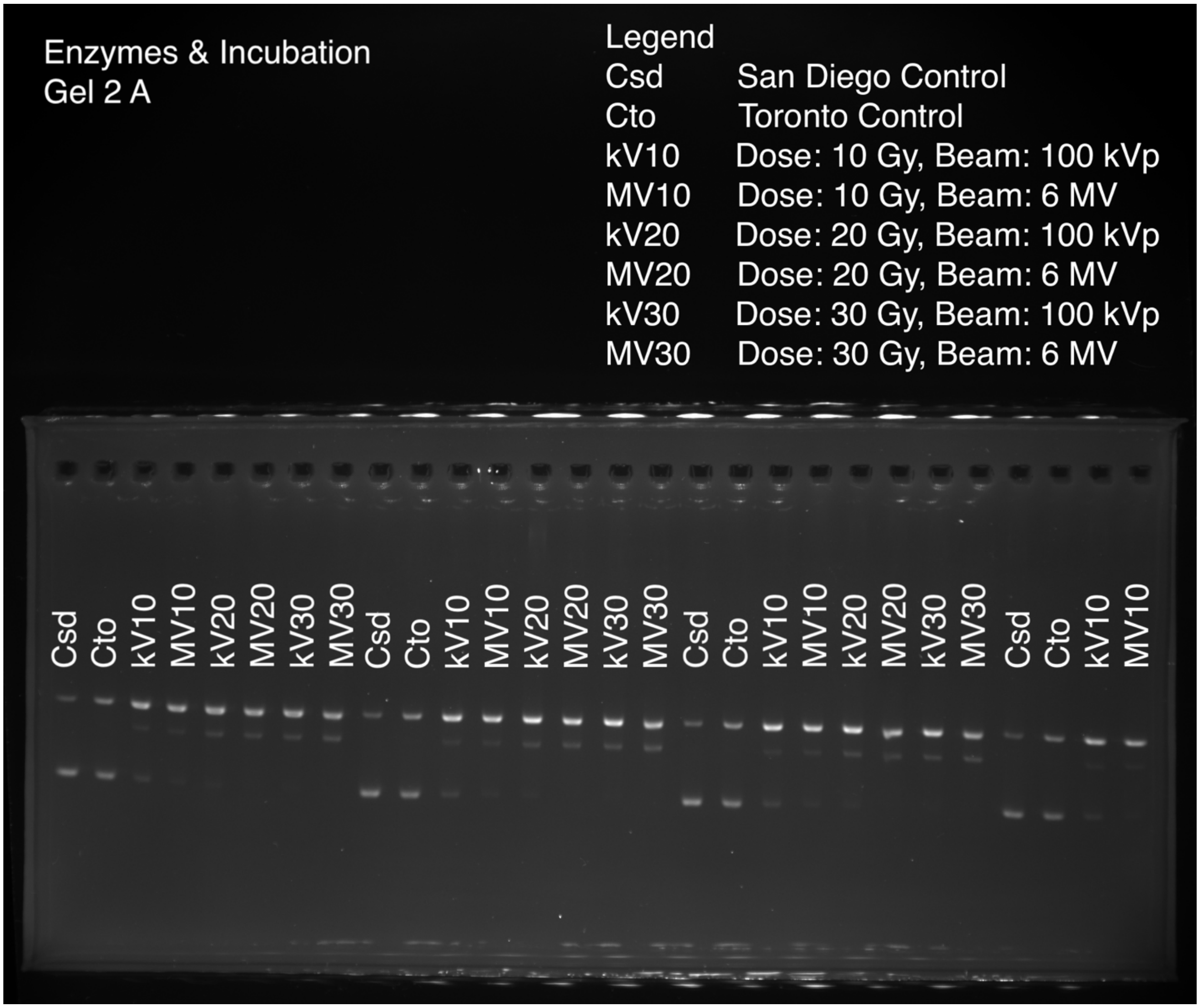
Enzymes & Incubation
Gel 2 A
Legend
Csd San Diego Control
Cto Toronto Control
kV10 Dose: 10 Gy, Beam: 100 kVp
MV10 Dose: 10 Gy, Beam: 6 MV
kV20 Dose: 20 Gy, Beam: 100 kVp
MV20 Dose: 20 Gy, Beam: 6 MV
kV30 Dose: 30 Gy, Beam: 100 kVp
MV30 Dose: 30 Gy, Beam: 6 MV
Csd Cto kV10 MV10 kV20 MV20 kV30 MV30 Csd Cto kV10 MV10 kV20 MV20 kV30 MV30 Csd Cto kV10 MV10 kV20 MV20 kV30 MV30 Csd Cto kV10 MV10

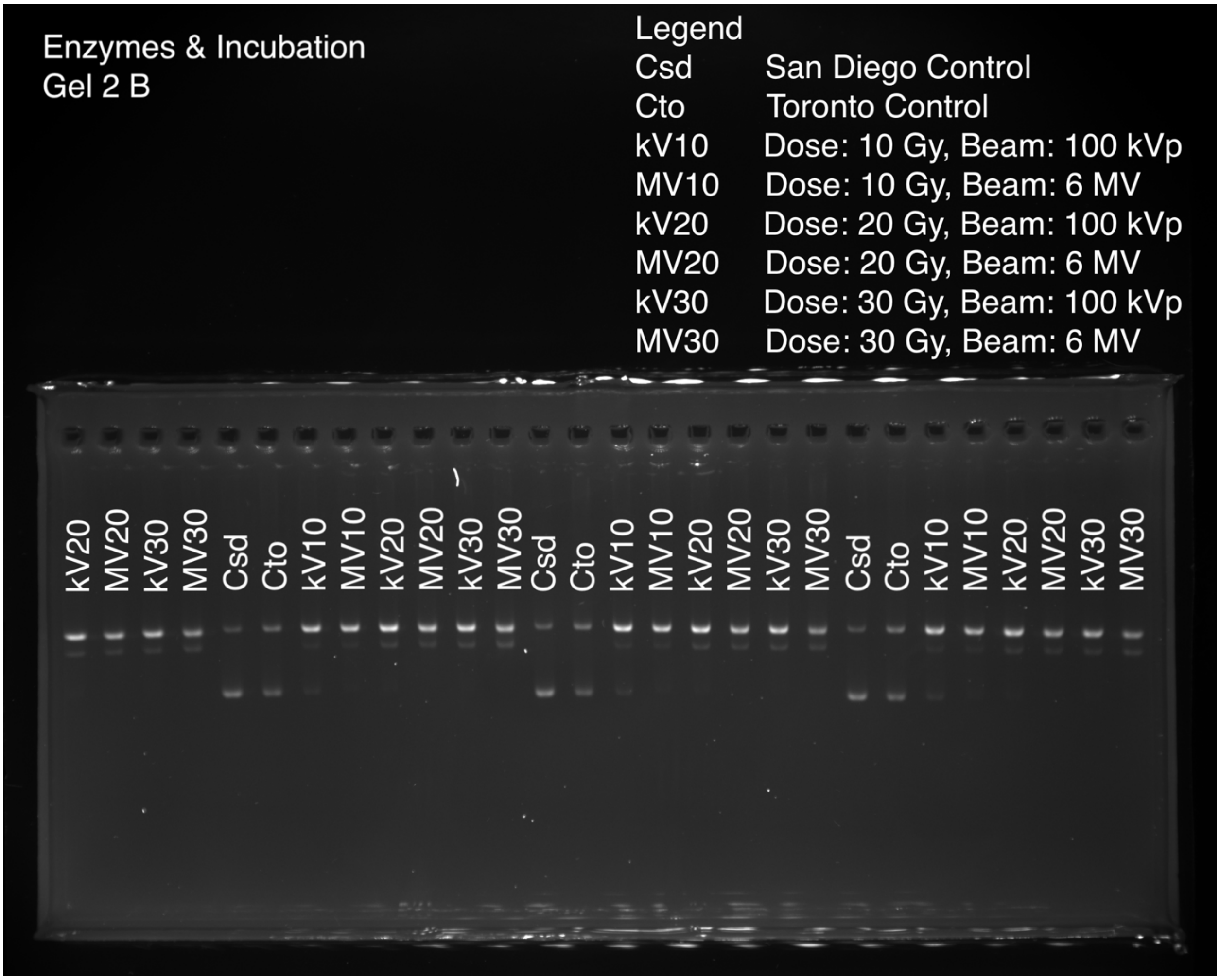
Enzymes & Incubation
Gel 2 B
Legend
Csd San Diego Control
Cto Toronto Control
kV10 Dose: 10 Gy, Beam: 100 kVp
MV10 Dose: 10 Gy, Beam: 6 MV
kV20 Dose: 20 Gy, Beam: 100 kVp
MV20 Dose: 20 Gy, Beam: 6 MV
kV30 Dose: 30 Gy, Beam: 100 kVp
MV30 Dose: 30 Gy, Beam: 6 MV
kV20 MV20 kV30 MV30 Csd Cto kV10 MV10 kV20 MV20 kV30 MV30 Csd Cto kV10 MV10 kV20 MV20 kV30 MV30 Csd Cto kV10 MV10 kV20 MV20 kV30 MV30
